# An *Aspergillus flavus* strain from bee bread of the Western honey bee (*Apis mellifera*) displays adaptations to distinctive features of the hive environment

**DOI:** 10.1002/ece3.10918

**Published:** 2024-02-22

**Authors:** Daniel S. Bush, Bernarda Calla, May R. Berenbaum

**Affiliations:** ^1^ Deparment of Entomology University of Illinois Urbana Illinois USA; ^2^ USDA‐ARS Pacific Shellfish Research Unit Corvallis Oregon USA

**Keywords:** aflatoxin, coevolution, fungus, insect, propolis

## Abstract

*Aspergillus* fungi are ubiquitous inhabitants of colonies of the western honey bee (*Apis mellifera*), where they interact with bees in associations ranging from parasitism to possible mutualism. *Aspergillus Flavi* fungi are frequently found in bee bread (pollen processed for longterm storage) and are thought to contribute to food preparation, processing, preservation, and digestion. Conditions in the hive are challenging for fungi due, in part, to xeric and acidic properties of bee bread and the omnipresence of propolis, an antimicrobial product manufactured by bees from plant resins. We used quantitative and qualitative assays to determine whether *A. flavus* isolated from bee bread demonstrates tolerance for hive environmental conditions in terms of temperature, pH, osmotic pressure, and propolis exposure. Comparisons made use of three strains of *A. flavus*: a fungal biocontrol product not known from beehives (AF36), a strain isolated from bee bread (AFBB) in hives from central Illinois, and a pathogenic strain from a honey bee colony displaying symptoms of stonebrood (AFPA). Strain AFBB displayed higher tolerance of acidic conditions, low matric potential (simulating xeric substrate), and propolis exposure than did other strains. A genomic comparison between this new strain and the reference NRRL‐3357 showed that AFBB, like AF36, might be blocked from carrying out aflatoxin biosynthesis. Sequence comparisons also revealed several missense variants in genes that encode proteins regulating osmotolerance and osmotic pressure in *Aspergillus* spp., including SakA, SskB, GfdA, and TcsB/Sln1. Collectively, results of our laboratory assays and genetic analyses are consistent with the suggestion that the strain isolated from bee bread is adapted to the bee bread environment and may have persisted due to a coevolutionary relationship between *Aspergillus* and *A. mellifera*. This finding bolsters recent concerns about the effects of fungicide use near bee colonies and broadens the ecological importance of highly adaptable fungal strains.

## Graphical abstract

*Aspergillus flavi* are frequently found in beebread (pollen processed for long‐term storage) and are thought to contribute to food preparation, processing, preservation, and digestion in the bee colony. We used quantitative and qualitative assays as well as genomic analysis to determine whether *A. flavus* isolated from beebread demonstrated tolerance for the hive environment in terms of temperature, pH, osmotic pressure, and propolis exposure. Both bioassays and molecular evidence suggest a history of coevolution between this strain of fungus and the western honey bee and a role for this fungus in bee food processing that could be disrupted by human activity.

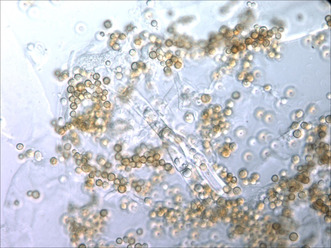

## 1 INTRODUCTION

As a perennial eusocial species, the western honey bee (*Apis mellifera* L.) stores large quantities of food in hives that represent abundant immobile sources of nutrition for microorganisms. The honey bee endo‐ and ecto‐microbiome, made up of a complex suite of bacterial and fungal species, is important in honey bee food preparation, processing, storage, and digestion (Anderson et al., 2013; Raymann, 2021). Honey bee researchers have long attempted to inventory local fungal diversity (“mycobiomes”) in honey bee colonies, referencing honey bee gut flora, flora from the gut and frass of the common intracolonial pest the wax moth (*Galleria mellonella*), and other locations in the hive (Callegari et al., 2021; Gilliam, 1997; Gilliam et al., 1974). In these studies, species in the nearly ubiquitous fungal genus *Aspergillus* comprise a disproportionately large part of the ectomycobiome. *Aspergillus flavus* and, to a lesser extent, *A. parasiticus* are of particular interest, as they produce highly carcinogenic aflatoxins and ochratoxins, which, apart from potentially adverse effects on honey bee health, can make bee products unsaleable (Amaike & Keller, 2011).

The relationship between honey bees and *A. flavus* is multidimensional and there is evidence that interactions between the two species range from adversarial to mutualistic (Becchimanzi & Nicoletti, 2022). *A. flavus* is one of the causes of the aspergillosis “stonebrood” (Foley et al., 2014; Seyedmousavi et al., 2015), but some strains may contribute to the suitability of the honey bee's processed food, notably, bee bread, via production of vitamins, enzymes, antimicrobial substances, lipids, and/or organic acids (Kieliszek et al., 2018). Such an association shares features with intimate associations between other hymenopterans and fungi, in which cultivated fungal mycelia grow on nutrient sources provided by the insect; examples include leafcutter ants (cf. Mueller et al., 2001) and even other bees, such as the Brazilian stingless bee *Scaptotrigona depilis* (Menezes et al., 2015).

The suggestion that honey bees and *A. flavus* may have a similarly lengthy and intimate coevolutionary history is supported by analogy with the relationship between *A. flavus* and *Aspergillus oryzae* (Ahlburg), a fungus involved in soy and sake fermentation that appears to be an ecotype of *A. flavus*, arising via human domestication (Payne et al., 2006). Certain aspects of hive conditions and the food sources themselves, however, are generally inhospitable to microbes. Environmental and competitive stress factors in corbicular pollen‐masses and bee bread make widespread fungal growth unlikely. Adverse abiotic properties include low water potential (i.e., xeric substrate), low pH, the presence of propolis, and the competitive pressure and secondary metabolites resulting from the abundance of acidophilic bacteria (Anderson et al., 2014).

Propolis is a resinous material used by honey bees for structural reinforcement, nest repair, and weatherproofing (Bankova et al., 2000; Ghisalberti, 1979). It is a mixture consisting of plant resins (from leaf buds, young leaves, twigs, or tree bark), honey, wax, and saliva. Propolis has extensive antimicrobial activity, including the inhibition of both bacteria and fungi (Bankova et al., 2000; Özcan, 1999). Some evidence exists that propolis inhibits the growth of *Aspergillus parasiticus* (Özcan, 2004) and prevents the production of aflatoxin B1 in *A. flavus* (Ghaly et al., 1998). Honey bees also use propolis to entomb hive invaders too large to remove from the hive after they are stung to death, to prevent corpse decomposition (Simone‐Finstrom & Spivak, 2010).

In addition to the abiotic and biotic stresses exerted by pollen‐masses and bee bread, the colony environment is characterized by year‐round temperatures higher than external ambient temperatures, all of which combine to present a distinct set of challenges for microbes in the hive environment to overcome. Irrespective of the nature of their ecological relationship with honey bees, strains of *A. flavus* likely require a high level of adaptation to coexist with honey bees. In this study, we set out to determine the extent to which members of *Aspergillus flavi* can withstand the distinctive environmental stresses associated with life in honey bee colonies. We isolated two new strains of *A. flavus* from beehives and used agarose, liquid minimal medium, and microplate assays to examine if these strains are adapted to the abiotic stresses found in pollen‐masses, bee bread, and the colony environment. In addition, we generated a partially annotated genome for one of our new bee‐associated *Aspergillus* strains and reviewed the genome for potential genetic signatures related to stress tolerance. We hypothesized that bee‐associated strains would display greater tolerance for colony‐specific environmental stresses and display altered gene sequences in regions related to aflatoxin production and stress tolerance in comparison with *A. flavus* reference strains.

## 2 MATERIALS AND METHODS

### 2.1 Obtaining and identifying fungal isolates

All fungal work was performed in a Purair safety Class II biosafety cabinet (Air Science, Page Park, FL). An atoxigenic strain of *A. flavus*, “AF36,” (NRRL 118543) originally recovered from cotton (Cotty, 1994) and under investigation as a possible biocontrol agent in the form of the registered formulation AF36 Prevail® (USEPA, 2017) was provided by T. Michailides, University of California‐Davis. This strain has no known association with honey bees, except incidentally during pollination in almond orchards in the California Central Valley, where honey bees provide managed pollination services during bloom periods (February through March).

**FIGURE 1:**
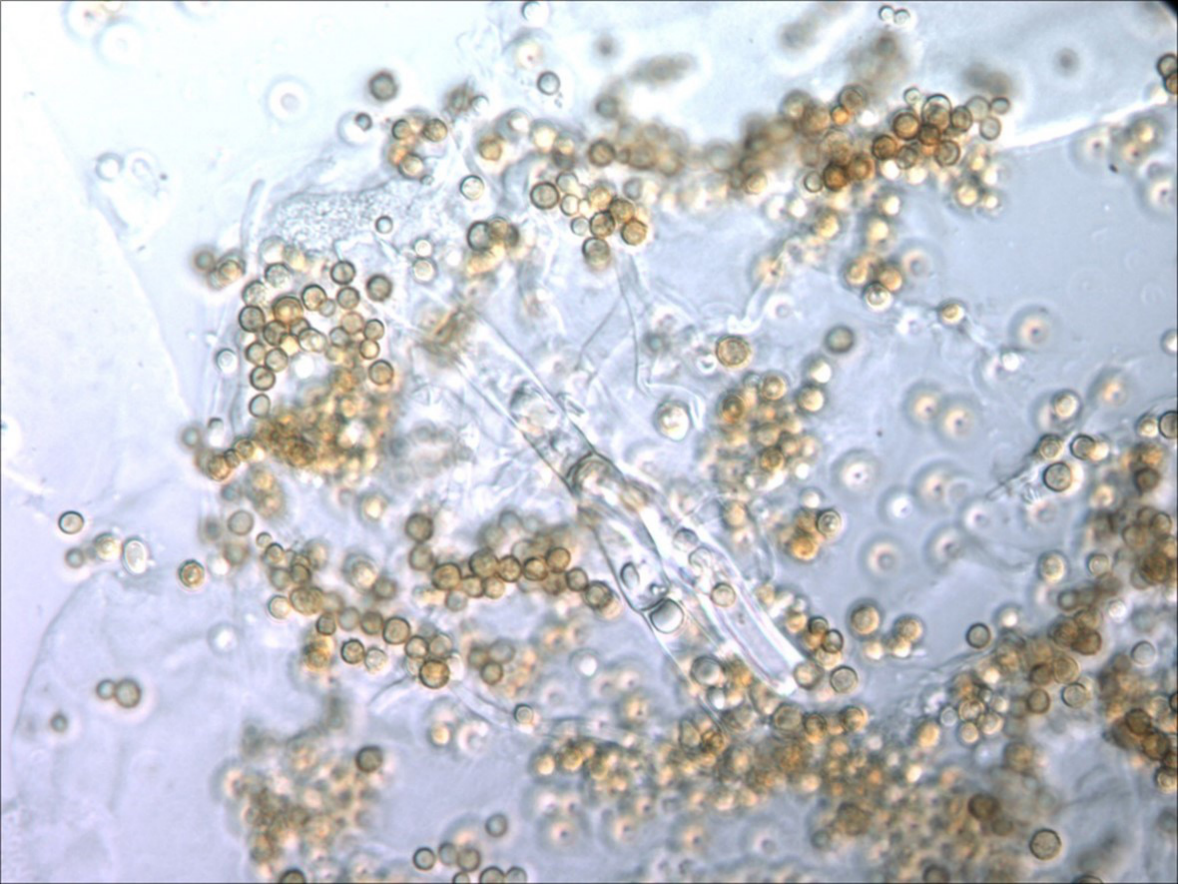
*Aspergillus flavus*. Microscopic image obtained with an Olympus BX51 microscope with differential interference contrast (DIC) equipped with an Olympus QColor 3 digital camera at 800×.

Samples of bee bread from UIUC experimental hives were brought back to the laboratory, diluted with tenfold serial dilution, and plated on potato dextrose agar (PDA, Sigma‐Aldrich, St. Louis, MO), which was incubated at room temperature in dark conditions and checked every 24 h for 1 week. We isolated fungal colonies and visually identified them (see Figure 1) at 800–2000× magnification with an Olympus BX51 microscope with differential interference contrast (DIC) and equipped with an Olympus QColor 3 digital camera. To confirm genus identification, we had the isolate sequenced with ITS1F‐ITS4. This process was performed with the help of Dr. Daniel Raudabaugh, using methods adapted from Raudabaugh et al. (2021). Briefly, DNA was obtained via NaOH extraction (Osmundson et al., 2012) from freshly ground mycelium. We conducted polymerase chain reaction (PCR) using a Bio‐Rad PTC 200 thermal cycler with a total reaction volume of 25 μL (12.5 μL GoTaq® Green Master Mix, 1 μL of each 10 μM primer ITSIF and ITS4, 3 μL Tris–HCl‐DNA extraction solution and 7.5 μL DNA free water). Next, we verified our product by gel electrophoresis (1% TBE agarose gel stained with ethidium bromide). The isolate was purified with a PCR Clean‐Up System (Promega). A BigDye® Terminator 3.1 cycle sequencing kit (Applied Biosystems Inc.) was used to sequence the ITS region, and Sequencher 5.1 was used to assist with quality assurance and base‐pair calls. Higher‐level taxonomic groupings were affirmed as in Raudabaugh et al. (2021) (and through visual inspection of conidia morphology), and species determination was made through sequence match using BLASTn against the NCBI (our thresholds for both genus and species identification were >85% query coverage with >93% ID (Wahl et al., 2018)). With the specific identity of the isolate confirmed, we started a colony of this strain, hereafter referred to as “AFBB.”

We also obtained a sample of fungus from bees in a private apiary experiencing a stonebrood outbreak (Mahomet, IL, GPS approx. 40.17, −88.41). Fungal samples were obtained via careful scraping from brood cells with visible infections (confirmed by knowledgeable colleagues at the UIUC Honey Bee Research Facility) performed with a sterile probe. Isolate identity was then confirmed based on morphological and molecular data as described. This isolate is referred to hereafter as “AFPA.”

### 2.2 Verifying strain uniqueness—Mating compatibility assays

To ascertain whether AFBB, AFPA, and AF36 represent separate strains, we used a method described by Smith (1962) with modifications to assess the compatibility of mating types. Smith's method takes advantage of the fact that changes at the mating gene loci of most ascomycete fungi occur at a dependable rate and result in mating and vegetative incompatibility at the subspecific level between strains. Self‐recognition between separate colonies of the same strain results in zones of growth avoidance to prevent competition. This method involves plating a plug of one strain of *A. flavus* on agarose (Sigma‐Aldrich) along with inoculate from either the same strain or one of the other two strains, repeating with all possible pairings, and observing growth patterns after 72 h.

### 2.3 Stress assays—Media

For liquid assays we maintained stocks of inoculate with a spore concentration of ~500 spores/mL. Spore concentrations were calculated using an improved Neubauer hemocytometer (Reichert, Buffalo, NY). Stocks of inoculate were stored at 32°C for up to 2 weeks in low‐light incubators. For any liquid suspensions used in this study, usually 0.1% Tween 80 (Sigma‐Aldrich) was added to prevent clumping. For each assay, we added 10 μL of inoculate. Unless indicated otherwise, liquid medium assays made use of a minimal nutrient medium, incubated at 25.5°C, and placed in a shaker at 180 rpm. The medium (Daniel Raudabaugh [Personal communication, June 1, 2017]) was made using the following recipe (per 1 L of medium): 6 g NaNO_3_, 0.52 g KCl, 0.52 g MgSO_4_·7H_2_O, 1.52 g KH_2_PO_4_, 10 g glucose. Sodium hydroxide was used to adjust pH before addition of glucose. Solid medium‐based assays were completed by transferring a small 2 mm × 2 mm agar plug containing actively growing mycelium onto the surface. The general solid growth medium was PDA incubated in low‐light conditions at 25.5°C.

#### 2.3.1 Temperature assays

We assessed *A. flavus* tolerance to elevated temperatures using both solid medium and liquid medium. We assayed our isolates at 30°C, 32°C, 34°C, 36°C, 38°C, and 40°C because beehives commonly reach internal temperatures of 35°C and higher (Oertel, 1949). For solid medium assays, plates were individually inoculated with one of the three strains, wrapped with Parafilm, and incubated at the target temperature in complete darkness. Fungal growth was measured by taking the mean diameter of the colony in three dimensions (Brancato & Golding, 1953) every ~24 h for 5 days. In the liquid medium assays, each strain was grown in Erlenmeyer flasks of liquid medium. After 5 days of incubation, we vacuum‐filtered the contents of each flask, lyophilized the fungal mass in a BUCHI Lyovapor L200 freeze‐dryer (BUCHI Corporation, New Castle, DE), and weighed the dry mass. This process was repeated five times for each strain with each treatment.

#### 2.3.2 pH assays

To perform qualitative assays for *A. flavus* pH tolerance, Petri dishes with PDA were adjusted to pH of 3, 4, 5, 6, and 7 with NaOH or HCl (Sigma‐Aldrich) and inoculated with fungus—three full sets of plates for each strain of *A. flavus—*and allowed to grow in an incubator at 25.5°C for 72 h, at which point they were visually inspected. Subsequently, the same pH concentrations were repeated in a liquid medium and incubated for 5 days, after which the fungal mass was isolated, dried, and weighed.

#### 2.3.3 Matric potential assays

A qualitative matric potential experiment was conducted to determine the tolerance of *A. flavus* to matric potential stress, which is a measure of the substrate's attraction for water. Liquid medium was prepared at a gradient of eight water potential levels (in increasing order of substrate attraction for water), 0, −5, −10, −20, −30, −40, −50, and – 60 Mpa, using polyethylene glycol (PEG) 8000 (FS) following the equation:

$$\psi =1.29{\left[\mathrm{PEG}\right]}^{2}T-140{\left[\mathrm{PEG}\right]}^{2}-4.0\left[\mathrm{PEG}\right]$$

where [PEG] = gram of PEG 8000 per gram of water and T = temperature (°C) (Michel, 1983; Raudabaugh & Miller, 2013). This assay was repeated three times for each strain, and all samples were incubated in these conditions for 14 days at 25.5°C and then visually assessed. After a visual assessment, the fungal mass was separated, dried, and weighed, as previously described.

#### 2.3.4 Propolis assays

*Aspergillus flavus* growth in the presence of propolis was evaluated with two sets of assays—one using solid medium and one using liquid medium. The PDA assays were adapted from Meneses et al. (2009). Half of the PDA plates contained a filter‐sterilized solution of propolis in 70% ethanol, originally from a 300 mg/L propolis suspension (Y.S. Organic Bee Farms, Sheridan, IL), and the other half contained only filter‐sterilized ethanol. Growth parameters were obtained as in temperature assays (see Section 2.3.1). This assay was repeated five times for each strain on each treatment. In the liquid medium trials, *A. flavus* of each strain was grown in Erlenmeyer flasks with and without 250 mg/L propolis. All inoculated media were incubated at 25.5°C. Separation, drying, and weighing of the fungal mass were conducted as described.

#### 2.3.5 Statistical methods

Two‐way Analysis of Variance (ANOVA) models in R v.4.1.1 (R Core Team, 2021; RStudio v.0.98.1083, R Foundation, Vienna, Austria) and post‐hoc comparisons were made using a Tukey's least square means procedure to determine statistically significant differences for the quantitative stress assays.

### 2.4 DNA extraction and sequencing

DNA extraction for AFBB was completed by following the protocol of Healey et al. (2014). A ZYMO Genomic DNA Clean and Concentrator‐25 kit (Zymo Research, Irvine, CA) was used to clean and concentrate the DNA. Libraries were prepared with the Hyper Library construction kit from Kapa Biosystems (Roche). Genomic DNA was sequenced on an Illumina MiSeq v.2 for paired‐end 250 nucleotide‐ long reads and in the Oxford Nanopore GridION (Oxford Nanopore Technologies, Oxford, UK) with the 48 h sequencing protocol.

#### 2.4.1 Genetic variant analysis

Illumina short‐reads were first used for a comparative analysis against two different *A. flavus* strains: the official, fully annotated *A. flavus* reference genome (NRRL3357, NCBI accession: JCVI‐afl1‐v3.0), and the *A. flavus* isolate AF36 (NCBI accession: ASM245617v2), used as a control in other assays in this study (Ehrlich & Cotty, 2004). Resulting Illumina sequencing reads were first assessed for quality by analyzing the output of MultiQC (Ewels et al., 2016). Based on that assessment, all forward and reverse reads were trimmed for the first 15 bases. Bases at the end of reads were trimmed if their Phred (phred33) score was below 36. In addition, reads with average quality scores below 33 were removed. All trimming was done with Trimmomatic v.0.32 (Bolger et al., 2014). Reads were mapped to each of the two genomes using bwa‐mem v. 0.7.17‐r1188 (Li, 2013). PCR duplicates, not properly paired, and low‐quality mapping reads were removed by first running samtools fixmate to fill in paired read attributes, then marking duplicates with samtools markdup and removing them. Mapped reads with mapping quality below 20 were filtered out (Li, 2011). Variants were called with bcftools (Li et al., 2009) after creating a mpileup file. The resulting .vcf files were inspected by plotting the available quality metrics individually to evaluate their distribution using R v.4.1.1. Based on that analysis, the variants were filtered according to two metrics: read depth (DP) at a variant position and mapping quality (MQ). Variants with DP greater than twice the mean DP across all the sets and variants with MQ smaller than 40 were removed. The set of filtered variants was then annotated using SnpEff v. snpeff‐4.5covid19‐1 (Cingolani et al., 2012).

#### 2.4.2 Genome assembly

The AFBB draft genome assembly was generated with paired‐end 250 nucleotide‐long reads from Illumina MiSeq v.2. and Oxford Nanopore reads using SPAdes hybrid assembler v.3.13.1 (Bankevich et al., 2012) with the following parameters: ‐k 21,33,55,77—careful—only‐assembler. Nanopore reads were first trimmed and filtered with NanoFilt with the options: ‐q 0 –headcrop 5 –tailcrop 3 –readtype 1D. Structural annotation for gene‐coding regions was done with GlimmerHMM v.3.4.0 (Majoros et al., 2004) as implemented by QUAST v. 5.0.2 (Gurevich et al., 2013). The completeness of coding sequences in the newly generated genome was evaluated with BUSCO v.3, with the fungal Ascomycota_odb9 database (Simão et al., 2015).

## 3 RESULTS

### 3.1 Identifying fungal isolates

**FIGURE 2:**
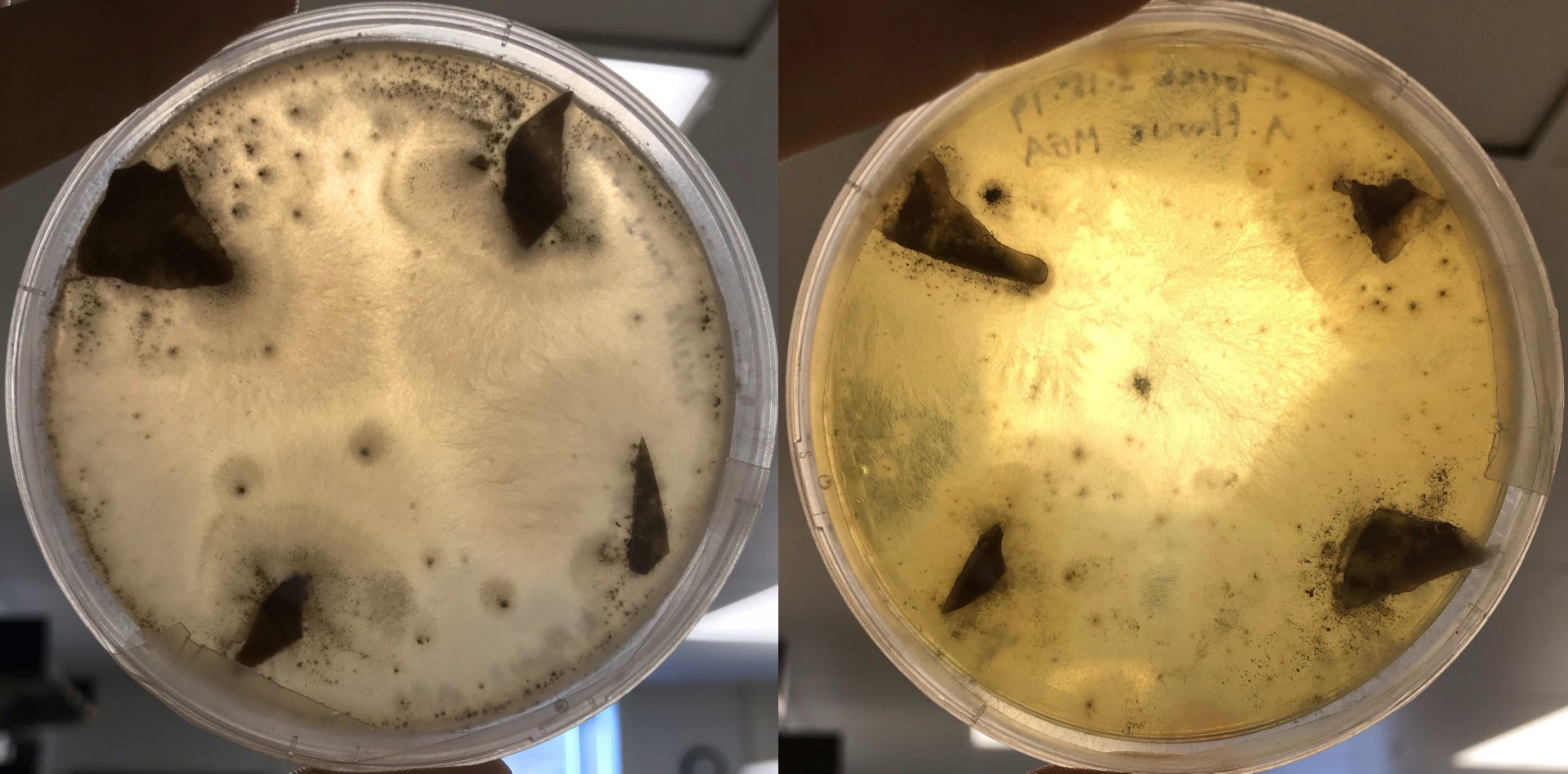
*Aspergillus flavus* colonies on PDA—strain AF36 only (L) exhibits zone of exclusion due to similar mating types; AF36 and AFBB (R) on the same plate do not.

The observed morphology of the isolates and results of ITS and BLASTn analysis demonstrated that the two isolates from bee colonies were *A. flavus*. In plates inoculated with only one strain, a clear zone of exclusion appeared, where hyphae did not grow due to the similarity of mating types. However, in all plates with two strains present, growth was unrestricted and undifferentiated (Figure 2).

### 3.2 Temperature, pH, matric‐stress, and propolis assays

No statistically significant differences were detected among the three strains in response to temperature. Growth rates held generally steady across all temperatures tested, typically reaching a diameter of slightly more than 40 mm by the end of solid medium assays and ~ 2.5 g by the end of liquid medium assays (data not shown).

**FIGURE 3:**
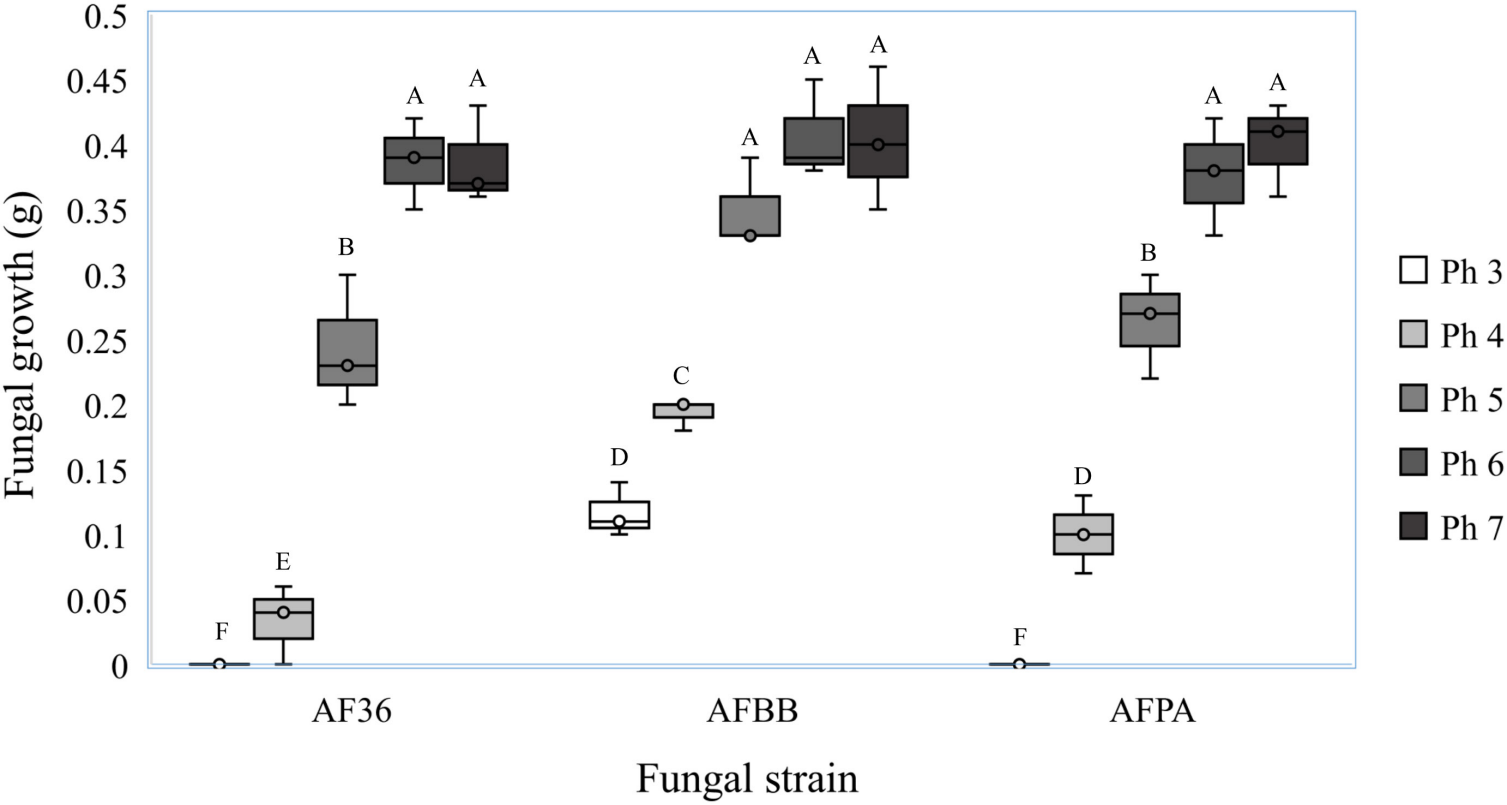
Mean dry weight of *Aspergillus flavus* not associated with honey bees (AF36), from bee bread (AFBB), and pathogenic on honey bees (AFPA) after 5 days in liquid minimal media calibrated to pH 3–7. Results with the same letter are not significantly different.

When the agarose plates were inoculated with AF36, AFPA, and AFBB, both hyphae and green conidia were observed on plates at pH 4 and higher on the AFBB plates within 7 days (Figure 3). AFBB colonies on the pH 4 treatment exhibited delayed growth relative to higher pH, with conidia appearing ~2 days later, on average. On plates at pH 3, AFBB exhibited visible growth, but colony size was smaller at each time point and its appearance was slightly discolored. Samples inoculated with AF36 and AFPA exhibited healthy growth patterns above pH 5, but they began to exhibit slower, discolored growth at pH 5; growth continued to decline at pH 4, and neither strain grew at pH 3. The pattern of strain response was similar on liquid minimal medium. There were statistically significant main effects for both pH (*F* = 84.66, df = 2, *p* < .001) and fungal strain (*F* = 6.09, df = 2, *p* < .05) after 5 days of incubation. At pH 5, AFBB already showed significantly more tolerance for acidic conditions than either AF36 (*t* = 3.81, df = 4, *p* < .05) or AFPA (*t* = 2.82, df = 4, *p* < .05). At pH 4, AFPA growth was intermediate, and this strain was slightly more tolerant to low pH than AF36 (*t* = 2.70, df = 4, *p* < .05). Neither AF36 nor AFPA produced any visible growth at pH 3 (Figure 3).

**FIGURE 4:**
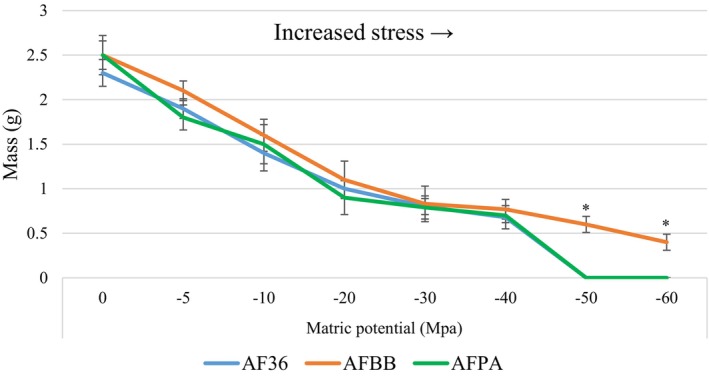
Mean dry weight (±SD) of *Aspergillus flavus* not associated with honey bees (AF36), from bee bread (AFBB), and pathogenic on honey bees (AFPA) after 14 days in liquid minimal media calibrated to matric potential of 0 to −60 Mpa. Asterisks denote significantly different results at the indicated level of matric potential.

AFBB exhibited significantly greater growth at low matric potential than did AF36 or AFPA, which did not even produce visible hyphae at −60 or −50 Mpa. However, from −40 to 0 Mpa there were no significant differences among the strains (Figure 4).

### 3.3 Propolis assays

**FIGURE 5:**
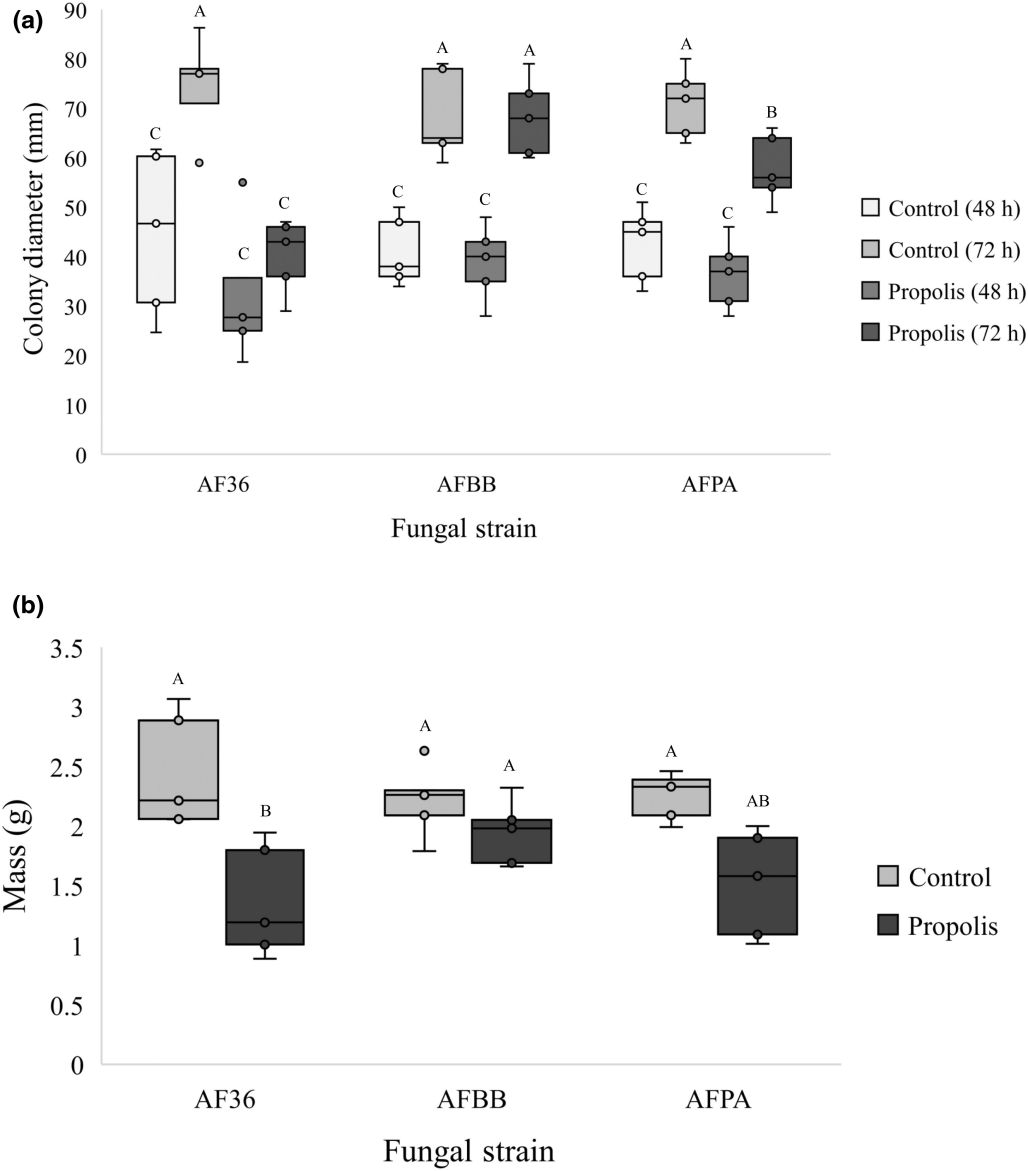
Mean growth of *Aspergillus flavus* colonies not associated with honey bees (AF36), from bee bread (AFBB), and pathogenic on honey bees (AFPA) after 48 and 72 h on agarose medium with and without propolis. Results with the same letter are not significantly different. Mean dry weight of *A. flavus* not associated with honey bees (AF36), from bee bread (AFBB), and pathogenic on honey bees (AFPA) after 5 days on liquid minimal medium with and without propolis. Results with the same letter are not significantly different.

On agarose medium, there was a significant negative main effect of propolis on fungal growth after 72 h (*F* = 16.14, df = 1, *p* < .001), although the overall effect of fungal strain was not significantly different across treatments. However, at 72 h, all three strains separated statistically according to the post hoc test (Figure 5). AFBB was more tolerant of propolis than AFPA (*t* = 1.98, df = 8, *p* < .05) or AF36 (*t* = 3.21, df = 8, *p* < .05), while AFPA exhibited slightly greater tolerance than AF36 to propolis (*t* = 1.99, df = 8, *p* < .05). In the liquid minimal media trials, propolis presence/absence had a significant overall effect (*F* = 9.94, df = 1, *p* < .05). Strain AFBB was more tolerant of propolis than AF36 at 5 days (*t* = 2.34, df = 8, *p* < .05), whereas the tolerance of AFPA was intermediate between AFBB and AF36 (Figure 5).

### 3.4 Variant analyses

Sequencing of Illumina libraries resulted in 14,303,365 read pairs. After trimming and filtering 75.63 pairs were retained (10,817.303 reads). Forward‐only surviving reads were 17.34% and reverse‐only surviving reads were 1.6%. Oxford Nanopore sequencing resulted in 2.6 million reads, with most of the reads between 1 kb and 15 kb and with the longest read around 50 kb. After filtering, 88% of the reads were retained. Mapping of the AFBB Illumina short reads to the AF36 genome was slightly better than to the reference NRRL 3357 (98.68% vs. 96.44% total mapped reads, respectively). We identified a total of 17,002 high‐confidence variants between AFBB vs. AF36. Of these, 427 were indels and the rest were SNPs. The majority of variants were located upstream of coding regions within genes (58.1%), or in intergenic regions (25.5%). Only one variant had a possible “high impact” according to snpEff analysis; this variant is an insertion located on a gene coding for a widely conserved, uncharacterized protein (gene ID in AF36: COH20_010776, with 93% similarity to XP_002372685.1 in NRRL 3357 reference).

Between AFBB vs NRRL 3357, we identified 178,908 high‐confidence variant sites with 12,507 INDELS. A large percentage of these variants were located upstream of coding regions (42.7%); there were also many variants downstream of coding regions (41.7%). Variants in intergenic regions accounted for 6.7%. There were 3.3% variants within exons, including exon losses, stop gains or losses, and missense variants (Appendix S1).

The most over‐represented gene ontology (GO) terms for genes affected by high‐impact variants between AFBB and NRRL3357 were glutamate biosynthetic process (Biological process; GO:0006537) and extracellular region (Cellular compartment; GO:0044421). Genes with high‐impact variants in the AFBB strain within these GO categories belong to amino acid metabolism, symbiosis, environment communication, environmental sensing, trafficking, and toxin production (Appendix S2: Table S1). Within the glutamate biosynthetic pathway, we identified three separate genes encoding pyrroline‐5‐carboxylate dehydrogenase that have high‐impact variants.

### 3.5 Genome assembly

To improve comparative power, we generated a draft genome assembly for AFBB using both the paired‐end 250 nt‐long reads from Illumina MiSeq v.2. and Oxford Nanopore reads using SPAdes hybrid assembler (Bankevich et al., 2012). Our AFBB draft genome assembly has a total length of 36 Mb in 222 scaffolds (N50 = 1,999,547). Glimmer predicted 19,198 unique mRNAs and 14,122 predicted peptide sequences in the assembly. Both genome size and number of predicted proteins agree with other *A. flavus* assemblies. The lack of continuity in the assembly is likely a result of the initial DNA, which was highly fragmented; several strategies failed to improve the resulting assembly. The BUSCO results with the fungi‐odb9 database with 85 species indicates that most genes were successfully assembled. Universal single‐copy ortholog statistics for this assembly are as follows: Complete: 100.0% Complete and Single: 99.3%, Complete and Duplicate: 0.7%, Fragmented: 0.0%, Missing: 0.0%, total BUSCO groups searched: 290.

### 3.6 Genetic signatures related to stress tolerance—Osmotolerance genes in AFBB

We searched for variants in AFBB genes that are part of the high osmolarity glycerol (HOG) pathway and identified 474 SNPs across 13 genes. Although no high‐impact SNPs (according to SNPEff classification) were found, there were 30 SNPs with low‐ to moderate‐impact, of which five were missense variants in the genes encoding SakA and SskB, MAP kinases known to regulate osmotolerance in *Aspergillus* spp. (Duran et al., 2010; Tumukunde et al., 2019). There were also SNPs in genes encoding a putative glycerol 3‐phosphate dehydrogenase (GfdA), and a putative sensor histidine kinase/response regulator TcsB/Sln1, both of which are regulated by the SakA and SskB MAP kinases in response to osmotic pressure (Appendix S2: Table S1). These variants were found when comparing AFBB with the reference NRRL‐3357; there were no variants with effects on these genes in AFBB using AF36 as reference.

### 3.7 Aflatoxin biosynthesis pathway

**FIGURE 6:**
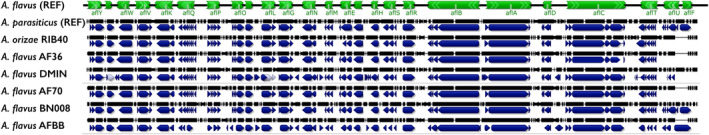
The aflatoxin biosynthetic pathway is *Aspergillus* spp. *A. flavus* REF is strain NRRL 3357 used as reference genome, and *A. parasiticus* REF is strain NRRL 5862 reference genome. The alignment produced by progressiveMauve (Darling et al., 2010) shows high synteny in a ~ 73 kb region. The reference *A. flavus* gene models are in green, for all other species and strains the black lines and dashes are the genomic DNA and blue arrows denote coding sequences. NCBI accession for each strain are as follows: *A. parasiticus* (AY371490.1); *A. oryzae* (AB196490.1); *A. flavus* AF36 (AY510455.1); *A. flavus* DMIN (ASM2365363v1); *A. flavus* AF70 (ASM95283v1); *A. flavus* BN008 (AY510452.1).

To evaluate the potential ability of AFBB to produce aflatoxin, the newly assembled AFBB genome was mined for the aflatoxin biosynthetic pathway. A gene cluster was found on a ~82 Kbp region in scaffold “Node 12” that contained the 23 core aflatoxin biosynthetic genes (Yu et al., 2004). All these genes are also present in NRRL 3357 and AF36 genomes. Synteny conservation and high sequence identity (>85% and up to 99.5%) were found when comparing this region in AFBB to five other published aflatoxin biosynthetic pathways in other *A. flavus* strains (Figure 6).

**FIGURE 7:**
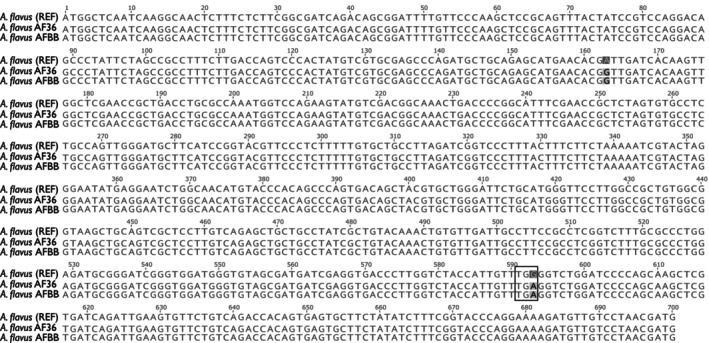
Region of the *Aspergillus flavus* polyketide synthase A (*pksA*) gene. The *A. flavus* strain NRRL 3357, used as reference, produces aflatoxin. *A. flavus* AF36 does not produce aflatoxin due to a mutation in *pksA* that leads to the TGA stop codon that truncates PksA rendering it non‐functional (Ehrlich & Cotty, 2004). We found the same mutation in AFBB.

In AFBB, one of the genes in the aflatoxin biosynthesis pathway, *pksA* (aka *aflC*), that encodes polyketide synthase has a nucleotide substitution G ➔A at nt 593 (Figure 7). This substitution was previously found in the non‐aflatoxigenic AF36 strain and is known to create a premature stop that truncates the synthesis of PksA in several atoxigenic *A. flavus* strains (Ehrlich & Cotty, 2004).

## 4 DISCUSSION

### 4.1 Response to environmental stress factors

In theory, the properties of bee bread should impose major stresses on microorganisms such as filamentous fungi attempting to colonize it as a habitat. It has very low pH, sometimes as low as 4, and exerts substantial osmotic pressure on fungi because of its low water potential (Herbert Jr. & Shimanuki, 1978). The overall results of the bioassays for the effects of propolis, pH, and matric potential are consistent with the hypothesis that the bee‐associated strains AFBB and AFPA, relative to the free‐living AF36 strain, display traits concordant with adaptation to the most distinctive abiotic characteristics of the beehive environment. High temperature does affect certain important metabolic processes and aflatoxin production in *Aspergillus* spp. (Oertel, 1949; Schindler et al., 1967), but, because typical bee colony conditions are within the suitable range for *A. flavus* (Hedayati et al., 2007), our experimental temperatures understandably had no noticeable effects on relative growth of the three strains. Notably, *A. flavus* is a widespread and fast‐adapting species, so attempts to characterize a general ideal temperature for all of our strains may be inaccurate. Depending on the details of the microhabitats in which conidia are typically found, it is possible AF36 could grow at temperatures even higher than the other two strains—this strain exists outside the beehive in areas of central California where soil surface temperature can exceed 55°C. Temperatures this high were not assessed in our study.

Bee bread is typically fairly acidic in nature. Acidophilic bacteria tolerate pH this low and abound in the gut microflora of honey bees and in bee bread (Anderson et al., 2013). Certain aspects of *A. flavus* metabolism and secondary chemical production are likely influenced by pH, including aflatoxin synthesis and production of sclerotia and pigments that protect against other environmental stresses (Chang et al., 2020; Cotty, 1988). Strain AFBB withstood extremely low pH and even produced few conidia at pH 3. It seems unlikely that low pH alone could prevent AFBB from growing in bee bread. Although the other two strains were technically able to grow at pH 4, they did not produce visible conidia, and the colonies were discolored to brown or red. This discoloration may be due to a lack of DHN‐melanin, an important conidial pigment that is reduced under stressful growth conditions (Pal et al., 2013), but further research is needed to confirm this connection. Because AFPA establishes itself within the bodies of larvae and pupae rather than within bee bread (composed largely of tissues from pollen), it presumably encounters different pH conditions. Capped honey bee brood have a hemolymph pH range of 6.60–6.65 (Bishop, 1923), and this strain is unlikely to encounter highly acidic conditions as a consequence of infecting the host, as might AFBB in bee bread.

Bee bread is a xeric substrate that exerts significant osmotic pressure on microbes attempting to colonize it. We used polyethylene glycol in an aqueous substrate in order to simulate pressure from low matric potential. All three strains of *A. flavus* showed a high degree of tolerance for osmotic pressure, but AFBB tolerated conditions of −60 Mpa. This is a very low matric potential that likely lends strain AFBB the ability to withstand great osmotic stress. Water availability and electrolyte balance are critical for fungal metabolism (Holmquist et al., 1983). *Aspergillus flavus* is ancestrally a soil fungus (Amaike & Keller, 2011), and AF36 is used for attempted biocontrol of toxigenic *A. flavus* strains in semi‐arid and Mediterranean agroecosystems in Arizona and California (Cotty, 1994); sandy soils and other substrates that would typically be considered inhospitable rarely reach matric potential below −20 Mpa (Chowdhury et al., 2011). Thus, the high tolerance for the osmotic pressure of bee bread is consistent with the ecology of *A. flavus*, although the tolerance of AFBB is higher than previously noted for *Aspergillus* species (Giorni et al., 2008; Nesci et al., 2004).

Propolis has known fungistatic properties that may help both to exclude harmful microbiota and promote beneficial bacterial flora in the colony (Dalenberg et al., 2020; Özcan, 2004). While the reduced growth of AF36 in the presence of even low concentrations of propolis is consistent with its fungistatic properties, the two bee colony strains responded to stress conditions differently. Strain AFBB showed significant tolerance for propolis in two different media, whereas AFPA displayed signs of tolerance in bioassays only on agarose. Either strain might encounter propolis via direct contact with the walls of the comb or via transfer from adult bees. Additionally, Niu et al. (2010) demonstrated a possible role for propolis in ameliorating the adverse effects of aflatoxins such as aflatoxin B1, produced by *A. flavus*, on bee health, by enhancing the activity of cytochrome P450 detoxification enzymes. The fact that there are signs of adaptation on both sides of this relationship suggests a possible history of coevolution in the dynamic between some *Aspergillus* isolates and honey bees.

### 4.2 Potential genetic signatures related to stress tolerance

Although the high fragmentation of the AFBB draft assembly prevented a full comparison between AFBB and other available *A. flavus* genome assemblies, we could identify a set of variants that could be consequential. There were 178,908 high‐impact SNPs found in the AFBB strain using the aflatoxin‐producing NRLL 3357 as reference. Using gene ontology enrichment, we saw that several specific biological pathways and cellular processes were significantly enriched for these variants in AFBB. Seen collectively, these processes are all associated with host–microbe interactions, suggesting that AFBB and NRRL 3357 differ primarily in how they interact with the hive environment.

Strain AFBB shares a polymorphism in the polyketide synthase gene seen in some other atoxigenic strains of fungi. Strain AF36, for instance, has a polymorphism in the gene *pksA*, altering a G to an A, which is sufficient to prevent all aflatoxin production; this is in addition to other amino acid changes in proteins encoded by *hexA* and *hexB* that involve biosynthesis of aflatoxin precursors (c.f. Ehrlich & Cotty, 2004). If AFBB is indeed atoxigenic—in addition to the substantial tolerance it displays toward multiple environmental stresses in the colony—it may have a lengthy evolutionary history with honey bees. This hypothesis is consistent with the suggestion that *A. flavus*, among other mold species commonly found in bee bread, produces enzymes associated with fermentation of bee pollen (Gilliam & Prest, 1989).

### 4.3 Parallel examples of co‐evolution among fungi and other bee species

There may be a parallel example of impacts of fungal domestication for food production by bees in the history of *A. flavus* domestication for food production by humans. Gibbons et al. (2012) described the origins of *A. oryzae*, a (possibly cultivated) ecotype of *A. flavus* now used for fermentation of food products such as sake and soy sauce, which could be seen as paralleling the process by which fermented food sources are processed and consumed in the beehive. The unusual resistance of honey bees to mycotoxins, especially aflatoxins, suggests a long‐standing coevolutionary relationship with *Aspergillus* species (Niu et al., 2010). Parish et al. (2020) reported that honey bees may even graze on fungal spores or conidia for nutritional benefits, which supports a hypothesis of coevolution. Other species of bees have associations with fungi, and interactions between bees and nonpathogenic fungi range from obligate mutualisms and grazing to complex effects on bee behavior (Menezes et al., 2015; Rutkowski et al., 2023).

There are explanations for these findings other than a hypothetical mutualism between honey bees and *A. flavus* that have not been addressed experimentally in this study. Mutualistic coevolution with the honey bee, e.g., is not the only possible explanation for reduced mycotoxin production, either in the short term or over evolutionary history. Heavy competition with other fungi, especially congeners, can also lead to reduced production of aflatoxin, apparently to divert resources from defense to enhanced growth (Ehrlich, 1987, Sarrocco et al., 2019, Schamann et al., 2021). Notwithstanding, the findings of this study suggest that indiscriminate usage of fungicides may lead to non‐target effects on fungi in the beehive environment that influence bee health (vanEngelsdorp et al., 2009; Yoder et al., 2017). Because bee bread and pollen are plant‐derived and also host many other organisms, further investigation of the genome of AFBB and bioassays using bacterial and plant secondary compounds or live bees could help elucidate the role of this strain in the rich microbiome of the *A. mellifera* colony.

## AUTHOR CONTRIBUTIONS

**Daniel S. Bush:** Conceptualization (equal); data curation (equal); formal analysis (equal); investigation (equal); methodology (equal); resources (equal); software (equal); validation (equal); visualization (equal); writing – original draft (equal); writing – review and editing (equal). **Bernarda Calla:** Conceptualization (equal); data curation (equal); formal analysis (equal); funding acquisition (equal); investigation (equal); methodology (equal); resources (equal); software (equal); validation (equal); visualization (equal); writing – original draft (equal); writing – review and editing (equal). **May R. Berenbaum:** Conceptualization (equal); funding acquisition (equal); project administration (equal); supervision (equal); writing – original draft (equal); writing – review and editing (equal).

## CONFLICT OF INTEREST STATEMENT

None declared.

## Supporting information


Appendix S1

Appendix S2

## Data Availability

DNA sequencing data are available at the NCBI SRA (https://www.ncbi.nlm.nih.gov/sra). Other data referenced in the appendices are separately deposited in the Illinois Data Bank, University of Illinois Library (https://databank.illinois.edu/) at the persistent link https://doi.org/10.13012/B2IDB‐7212497_V1. NCBI access numbers for the fungal genome sequence in this paper are as follows: SubmissionID: SUB11750690; BioProject ID: PRJNA857094.
